# Modular Point-of-Care Breath Analyzer and Shape Taxonomy-Based Machine Learning for Gastric Cancer Detection

**DOI:** 10.3390/diagnostics12020491

**Published:** 2022-02-14

**Authors:** Inese Polaka, Manohar Prasad Bhandari, Linda Mezmale, Linda Anarkulova, Viktors Veliks, Armands Sivins, Anna Marija Lescinska, Ivars Tolmanis, Ilona Vilkoite, Igors Ivanovs, Marta Padilla, Jan Mitrovics, Gidi Shani, Hossam Haick, Marcis Leja

**Affiliations:** 1 Institute of Clinical and Preventive Medicine, University of Latvia, LV-1586 Riga, Latvia; manohar_prasad.bhandari@lu.lv (M.P.B.); linda.mezmale@lu.lv (L.M.); linda.anarkulova@lu.lv (L.A.); viktors.veliks@lu.lv (V.V.); armands.sivins@aslimnica.lv (A.S.); lescinska@hotmail.com (A.M.L.); marcis.leja@lu.lv (M.L.); 2 Riga East University Hospital, LV-1038 Riga, Latvia; igors.ivanovs@lu.lv; 3 Faculty of Medicine, University of Latvia, LV-1586 Riga, Latvia; 4 Liepaja Regional Hospital, LV-3414 Liepaja, Latvia; 5 Faculty of Residency, Riga Stradins University, LV-1007 Riga, Latvia; 6 Digestive Diseases Center GASTRO, LV-1079 Riga, Latvia; ivars.tolmanis@gastrocentrs.lv (I.T.); ilona153@inbox.lv (I.V.); 7 Department of Internal Diseases, Riga Stradins University, LV-1007 Riga, Latvia; 8 Department of Doctoral Studies, Riga Stradins University, LV-1007 Riga, Latvia; 9 Health Centre 4, LV-1012 Riga, Latvia; 10 JLM Innovation GmbH, D-72070 Tübingen, Germany; marta.padilla@jlm-innovation.de (M.P.); jan.mitrovics@jlm-innovation.de (J.M.); 11 Laboratory for Nanomaterial-Based Devices, Technion—Israel Institute of Technology, Haifa 3200003, Israel; gshani@technion.ac.il (G.S.); hhossam@technion.ac.il (H.H.)

**Keywords:** gastric cancer, breath analysis, electronic nose, machine learning, screening

## Abstract

Background: Gastric cancer is one of the deadliest malignant diseases, and the non-invasive screening and diagnostics options for it are limited. In this article, we present a multi-modular device for breath analysis coupled with a machine learning approach for the detection of cancer-specific breath from the shapes of sensor response curves (taxonomies of clusters). Methods: We analyzed the breaths of 54 gastric cancer patients and 85 control group participants. The analysis was carried out using a breath analyzer with gold nanoparticle and metal oxide sensors. The response of the sensors was analyzed on the basis of the curve shapes and other features commonly used for comparison. These features were then used to train machine learning models using Naïve Bayes classifiers, Support Vector Machines and Random Forests. Results: The accuracy of the trained models reached 77.8% (sensitivity: up to 66.54%; specificity: up to 92.39%). The use of the proposed shape-based features improved the accuracy in most cases, especially the overall accuracy and sensitivity. Conclusions: The results show that this point-of-care breath analyzer and data analysis approach constitute a promising combination for the detection of gastric cancer-specific breath. The cluster taxonomy-based sensor reaction curve representation improved the results, and could be used in other similar applications.

## 1. Introduction

Gastric cancer ranks fourth among the most common cancers worldwide. It shows a lack of specific symptoms in the early stages, and is commonly characterized by late diagnosis, poor prognosis, and likely relapse [1]. The early detection of gastric cancer is the first step prior to diagnosis and screening for the reduction of mortality. However, there is no quick, reliable, non-invasive, and economical screening tool for gastric cancer. At present, upper gastrointestinal endoscopy with biopsy is the gold standard for the diagnosis of the condition, but this operation is time-consuming, expensive, and invasive; patient compliance is poor, and the demands on medical staff and equipment are typically high. Therefore, it is unaffordable for mass screening, and cannot provide the early diagnosis of gastric cancer.

Breathomics is a branch of metabolomics that helps us to diagnose diseases by analyzing volatile metabolites produced by changes in the metabolic processes caused by the disease [2,3]. So far, breath analysis has proved successful for the diagnosis of lung cancer, breast cancer, gastric cancer, prostate cancer, colorectal cancer, ovarian cancer, head-and-neck cancer, bladder cancer, and kidney disease [4,5]. The following have been used for the detection of gastric cancer: gas chromatography–mass spectrometry (GC-MS), the laboratory-based analysis of collected air, and some on-line analysis tools which are under development. There are known systems for the detection and identification of volatile organic compounds (VOCs) in exhaled breath using an array of sensors which are reactive to those VOCs.

Sensor-based exhaled breath analysis has shown significant promise for early gastric cancer detection in scientific and clinical practice because of its high accuracy, low cost, non-invasiveness, and ease of operation [6,7,8,9,10,11]. The application of such a diagnostic method is important in helping to reduce gastric cancer mortality. Its performance relies on its accuracy, sensitivity, specificity, and predictive values [4,12]. With advances in technology, the breath analysis approach could hold the key to the detection of gastric cancer, providing broader information on the progress of the disease, VOCs unique to gastric cancer, their origin, and the underlying biochemical mechanisms.

Various sensor-based electronic noses have been used in recent years to complement GC-MS for the detection and differentiation of various types of cancers, including gastric cancer [13,14,15,16,17]. Different self-powered respiration sensors and wearable biosensors are also used for non-invasive chemical breath analysis and physical respiratory motion detection [18,19,20,21,22]. These can monitor human respiratory patterns and behaviors spontaneously. GC-MS techniques are generally time-consuming and costly, and are not feasible for use in daily medical practice, whereas electronic noses can detect low concentrations of complex VOCs emitted from various matrices without necessarily identifying the individual volatile metabolites [23].

In this work, we report the diagnostic performance of a modular point-of-care breath analyzer with gold nanoparticle (GNP) and two different types of metal oxide (MOX) semiconductor sensors for the detection and identification of gastric cancer in an online mode that requires no additional breath collection procedures or laboratory settings. The proposed device was built on the basis of previous studies in laboratory settings [24], measurement reproducibility studies [25,26], and population studies [16] by improving the sensors and adding modules with other sensors to obtain more information from breath. Furthermore, we describe and validate a machine learning algorithm for data analysis that is used to classify gastric cancer cases and healthy controls, and we offer further recommendations to improve the device. The satisfactory performance of the developed model shows that the breath analyzer device could be a promising tool for the detection and classification of gastric cancer in a point-of-care clinical setting. It holds potential for future clinical application as a fast, non-invasive, and well-directed method for gastric cancer detection and screening.

## 2. Materials and Methods

### 2.1. Ethics

The study was approved by the Ethics Committee of the Riga East University Hospital Support Foundation (approval No. 18A/19). All of the participants provided signed consent before entering the study.

### 2.2. Study Participants

Patients who had reached at least 18 years of age and were able to undergo a breath exam were included, and signed a consent form. The following exclusion criteria were applied:

- known active malignant diseases other than gastric cancer,
- ongoing neoadjuvant chemotherapy,
- a history of stomach surgery (except vagotomy and ulcer suturing),
- inflammatory bowel disease (Crohn’s disease and ulcerative colitis),
- end-stage renal insufficiency,
- diabetes mellitus type I,
- active bronchial asthma, and
- a history of small bowel resections.

Patients with morphologically confirmed gastric adenocarcinoma were included in the study prior to their gastric surgery. A control group (patients without gastric cancer) was included prior to upper endoscopy. Histological reports were assessed after the examination, and the final grouping was performed after that.

After their inclusion in the study, the subjects were given specific instructions to follow before the measurements were made in order to minimize the effects of any strong and potentially interfering VOCs. They were informed by telephone or in writing at least 24 h prior to the planned procedure in order to ensure compliance with the requirements. The instructions included the following restrictions:

- fast for at least 12 h;
- refrain from drinking coffee, tea and soft drinks for at least 12 h;
- refrain from smoking for at least two hours;
- avoid alcohol for at least 24 h;
- do not clean your teeth within two hours before the procedure (no brushing, no mouthwash, no flossing if the floss has any aroma);
- avoid chewing gum and using any mouth fresheners for at least 12 h;
- refrain from using cosmetics/fragrances on the day of the test prior to the procedure;
- avoid excessive physical activity (the gym, jogging, cycling, intense physical work) for at least two hours prior to the test.

### 2.3. Breath Measurement

The study participants were invited to Latvia Oncology Centre, Riga East University Hospital (Riga, Latvia), where a separate room was designated solely for breath sample measurements in order to reduce the risk of contamination with other VOCs. The control and cancer group subjects were included randomly during the same time period (starting November 2019). A lifestyle and medical history questionnaire, including potential confounding factors, was completed prior to the breath sampling. After the questionnaire, the participants exhaled into a table-top device that analyzed their breath immediately after the exhalation.

**Figure 1:**
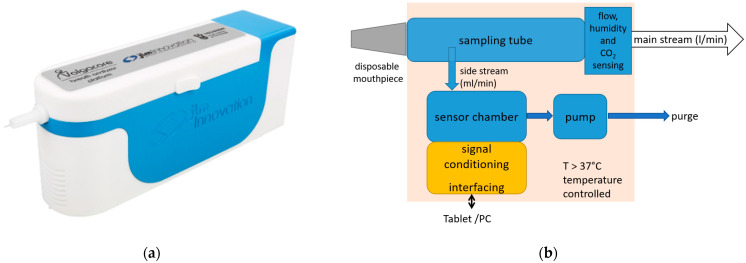
The point-of-care device used in the study: (**a**) the design of the device, with a disposable mouthpiece inserted at the front; (**b**) the main blocks of the system.

The point-of-care breath analyzer used in this study was a modular breath analyzer prototype developed by JLM Innovation GmbH (the device is shown in Figure 1a; the main blocks of the system are shown in Figure 1b). The main parts are: (i) a sampling unit that collects the exhaled air and monitors the air flow, which includes a pump that draws the alveolar air from the tube at the exhalation end to provide buffered-end tidal sampling; (ii) environment control sensors (temperature measurement for temperature control, and humidity and air pressure sensors); and (iii) a sensor chamber that holds three modules of sensors: one with eight GNP sensors developed by Technion (Israel Institute of Technology, Haifa, Israel), another with eight analogue MOX sensors, and a third with ten digital MOX sensors. The sensor set comprises monolayer-capped, organic, functionalized GNP sensors and chemoresistive analogue and digital surface mount devices (SMD). This heterogeneous array of sensors with different transducing mechanisms was specifically selected for the detection of gastric cancer on the basis of previous study results. The sensors were designed to be selective towards a wide range of VOCs in exhaled breath, including nonpolar and aromatic VOCs, and metabolites linked to intermolecular interactions, including Lewis acid-base, hydrogen bonding, and dipolar interactions.

The GNP sensors were functionalized with eight organic ligands to enhance their affinity for gastric cancer VOCs. These ligands were selected from a reservoir of 40, following a previous study carried out on more than 1000 clinical breath samples [24]. The organic ligands used to cover the GNPs for the detection and classification of gastric cancer from exhaled breath were: decanethiol, 2-ethylhexanethiol, 2-nitro-4-trifluoro-methylbenzenethiol, octadecanethiol, tert-dodecanethiol, 2-amino-4-chlorobenzenethiol, 2-mercaptobenzimidazole, 3-ethoxythiophenol, and 2-naphthalenethiol. The analogue and digital MOX sensors have broad sensitivities to the VOCs from exhaled breath. These sensor modules are designed to detect a wide range of VOCs, including ethanol, acetone, H_2_, ethane, isoprene, CO, nitrogen oxides (NOx), volatile sulfur compounds, CO_2_, and breath VOC mixtures.

In order to take a measurement, the device was connected to a computer and the measurement process was controlled from a computer-based graphical interface. Before the measurement began, medical personnel entered metadata, and a sample of the room air was taken and measured automatically to provide data from the sensor response to any potential background VOCs. Then, the patient was notified to prepare for exhalation by a message on the computer screen and flashing lights on the device. The patient exhaled into the device, and the last part of the breath was pumped into the sensor chamber and analyzed. The metadata and the sensor responses to the room air and the exhaled air were saved in a single JSON file on the computer.

### 2.4. Data Analysis

**Figure 2:**
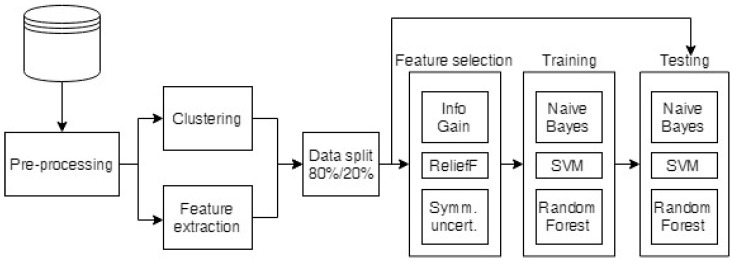
Diagram of the data analysis process.

The raw breath measurement data were first preprocessed to exclude any faulty measurements, equalize the lengths of the measurements, and clean the data, as described in Section 2.4.1. The preprocessed data were then used to build cluster taxonomies (see Section 2.4.2) and to extract the standard features for comparison (the minimum, maximum, average, area under the curve and the mean of the end (stable) part of the measurements). Then, the cluster taxonomies and the extracted features were run through feature selection algorithms to select the most informative attributes, which were classified as described in Section 2.4.3. The whole process is depicted in Figure 2.

#### 2.4.1. Preprocessing of the Raw Data

Before the sensor response curves were processed, measurements that lacked some of the sensor readings owing to technical problems were excluded from the analysis (ten gastric cancer patient and two control group measurements). Then, the measurements (one time series per sensor) were preprocessed by equalizing the measurement lengths (some sensors had extra time points at the beginning or end of the measurement because of the measurement specifics) such that they could be analyzed using methods that require equal-length time series. The readings were normalized against the final values of the baseline measurements (the room air measurements before the breath analysis) when the sensor response had stabilized in order to remove the effects of the environmental air and the VOCs present in it. All of the measurements were also examined in order to identify outliers by projecting the observations to the principal components, calculating orthogonal and score distances, and evaluating them as proposed by Rodionova and Pomerantsev [27]. The median filter was applied to reduce any noise in the sensor readings.

**Figure 3:**
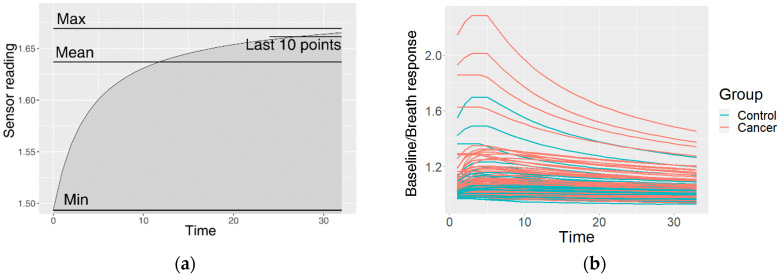

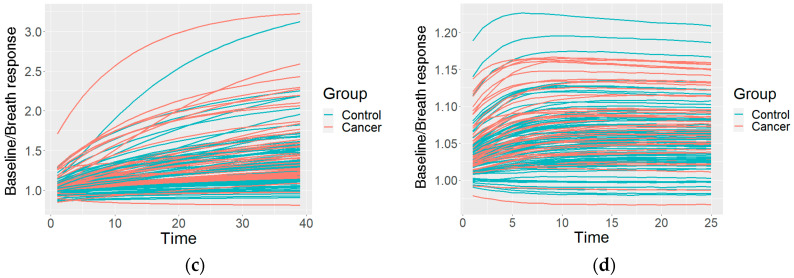
An example of the data after the preprocessing: (**a**) common features describing the curve; (**b**) curves of a GNP sensor; (**c**) curves of an analogue MOX sensor; (**d**) curves of a digital MOX sensor.

This resulted in one preprocessed time series of the sensor reactions to the breath for each sensor type. The GNP, analogue MOX, and digital MOX sensor responses (and common features) are shown in Figure 3.

The preprocessing was carried out in order to prepare the sensor response curves in the classification model training. They are usually analyzed using the features of the curve, including the steady-state response and transient responses such as the minimum, mean or maximum values, or the area under the curve [28,29]. However, this approach removes a lot of potential information in the curve. Therefore, in this study, we used the shape of the curve as a feature. In order to describe the curve shape, we used cluster-specific curves. Clustering divides observations into groups so that the observations within a group are more similar to each other than to those from other groups. Therefore, similar curves were grouped into clusters, and membership of this cluster (measurements similar to the mean cluster curve) was used as a feature. The clustering process is described in the following subsection. Feature extraction and selection are important for the understanding of the data by ranking the features on the basis of their contributions to the classification. Therefore, for the given observations, we can infer which are the best sensors and the best feature extraction method.

#### 2.4.2. Clustering of the Measurements

**Figure 4:**
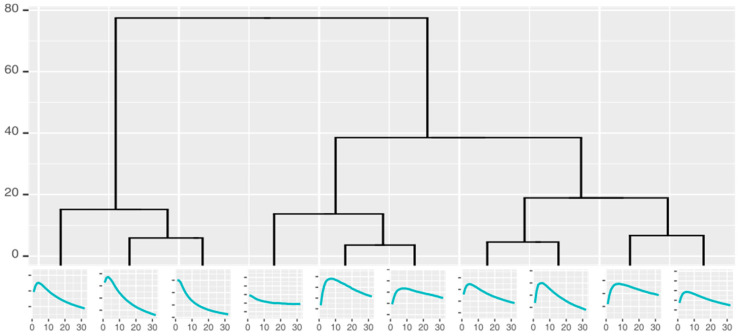
Cluster taxonomy of the responses from one gold nanoparticle sensor.

Curve shape taxonomies holding the curves that can potentially be used for classification were built by repeatedly clustering the measurement curves into k similar groups and identifying the characteristic shape of each cluster. The number of the groups could not be defined precisely before the analysis; as such, we used hierarchical clustering and cuts in the resulting dendrograms to generate 2–10 clusters, all of which were used to create a hierarchical taxonomy (Figure 4) of the groups (represented by the characteristic curves).

The clustering was performed by the hierarchical agglomerative method, using dynamic time warping (DTWARP) and Euclidean distances, each being tested by two different linkage methods: complete and Ward linkage.

Euclidean distance was chosen because it is the most common distance metric. The distance *d* is calculated by comparing the distances of every two points (out of the total length of the times series *n*) of the two time-series (*TS*1 and *TS*2):

(1) $$d\left(TS1,TS2\right)=\sqrt{\sum_{i=1}^{n}{\left(TS1_{i}-TS2_{i}\right)}^{2}}$$

DTWARP [30] was used because it has proven effective in time-series clustering [31,32]. One of its strengths is matching values at different time points, which is also important in sensor response analysis because the reactions can occur at different speeds, and there can be delays before a reaction starts or before it reaches its maximum. DTWARP can be considered to be an extension of Euclidean distance that creates a mapping of points from two time series in order to minimize the pairwise Euclidean distance, such that each point is aligned with one or more points from the other time series.

The linkages chosen for this study have previously shown their strength in the creation of clusters with more similar sizes, seldom creating small clusters with a few data points that are further away from other points on the attribute axes but are not considered outliers. Ward’s distance minimizes the variance of data points in the clusters, while the complete distance considers the maximum distance between two clusters.

#### 2.4.3. Classification

The results of the clustering (hierarchy, group memberships, and the characteristic curves) representing the naturally-occurring groups of sensor responses were then used as input data in classifier induction to discriminate between the control and cancer classes. The membership of each breath to a cluster (at each level in the taxonomy) was used as a separate attribute. Different approaches can be used to determine the optimum number of clusters and the necessary cut in the taxonomy. Some classification algorithms have built-in feature selection procedures (such as rule- or tree-based classification algorithms). If the method of choice lacks a built-in option, the cut can be determined on the basis of expert choice, distances among clusters and other dissimilarity evaluation metrics, or feature selection algorithms. In our study, we chose the feature selection approach to ensure the best cluster sets for classification. In order to find the best cuts, we applied the following feature selection algorithms: Information Gain, ReliefF, and symmetrical uncertainty. Information Gain (also known as Kullback-Leibler divergence [33]) is a metric for the assessment of the reduction in entropy if a feature is used to create subgroups of data. It is used in many applications even though it does not take account of potential dependencies among features. ReliefF [34,35] can detect conditional dependencies between features, and can evaluate those features on the basis of how well they distinguish between similar instances. Symmetrical uncertainty assesses the correlation between a feature and the class, and was proposed as a robust filter by Yu and Liu [36].

The selected features were then used in classification (the steps of the data analysis process are given in Figure 2). The data were used to build Naïve Bayes (NB) classifiers, Support Vector Machines (SVM) and Random Forests (RF) using the algorithm implementations in the Weka libraries [37]. The NB [38] classification algorithm is a probability-based algorithm that calculates the conditional probabilities of classes from feature values, assuming no dependencies among the features. The SVM [39] algorithm constructs a hyperplane that separates the two classes and has the maximum margin (distance from the data points). RF [40] randomly constructs a number of decision trees and uses majority voting to predict the class.

NB and SVM are popular algorithms which are often applied in electronic nose-related machine learning tasks [41], and each is based on a different approach (probabilistic vs. function-based). The RF algorithm is an ensemble of tree classifiers, and should therefore be more accurate for the handling of more complex data, where simpler probabilistic or function-based algorithms would be unable to describe the relationships within the data.

Each dataset (common features and cluster taxonomies as features) was run through the feature selection, classification model training and testing cycle 1000 times. Each run was evaluated using metrics such as overall accuracy (the total percentage of correct predictions), sensitivity (the percentage of the correctly identified cancer group), specificity (the percentage of the correctly identified control group) and the area under the ROC curve (AU-ROC). The accumulated data were used to calculate the mean values and the 95% confidence intervals (95% CI) of the results. The statistical significance of the differences was assessed using ANOVA with Bonferroni post-hoc analysis.

## 3. Results

The different sensor types gave very different results when analyzed separately. Gold nanoparticle sensors showed high overall accuracies, up to 77.61%, but their sensitivity did not surpass 55% (their specificity was 89%). The overall accuracy of analogue MOX sensors reached 69.94%, but their sensitivity was low (46%), and their specificity reached 83%. The overall accuracy of the digital MOX sensors was up to 67.88%, with rather low sensitivity (47%) and specificity (79%).

When all of the sensor modules were used for the classification, the results showed that the overall accuracy of the most accurate classification algorithm (Naïve Bayes) was over 70% (at best 77.81%), with sensitivity ranging from 46.9% to 66.54%, and specificity ranging from 83.64% to 85.27% depending on the features selected (see the best results in Table 1). The area under the ROC curve was from 0.774 to 0.817 (the ROC for these feature subsets, and all of the accuracy metrics for different combinations, are provided in Table S1 in the Supplementary Information).

The use of cluster taxonomies improved the overall accuracy (~5 percentage points) and sensitivity (~10 percentage points) and AU-ROC, while the specificity remained similar to the specificities obtained using the more common features.

**Figure 5:**
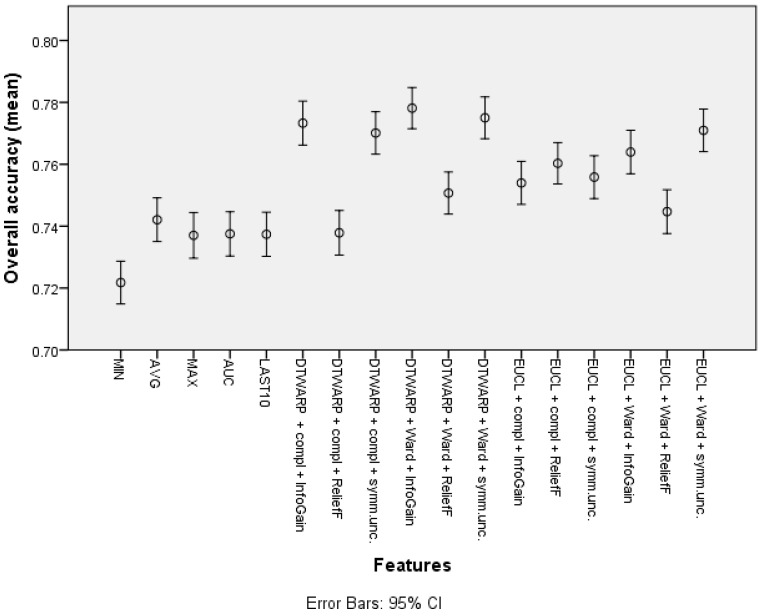
The overall accuracy of Naïve Bayes classifiers: mean values and 95% confidence intervals.

The overall accuracy (the mean values and 95% confidence intervals are shown in Figure 5) in the common feature datasets was mostly ~73.7% (95% CI: ~73.0–74.4%), with the best overall accuracy being shown by the average signal level, which was 74.21% (73.5–74.91%). When clusters were used instead of common features, the overall accuracy improved. The data subsets where the ReliefF feature selection approach was used showed the worst results in almost all of the distance and linkage combinations. The best overall accuracies were achieved in the datasets where the clusters were obtained using DTWARP distances. All of them were significantly better than the common features.

**Figure 6:**
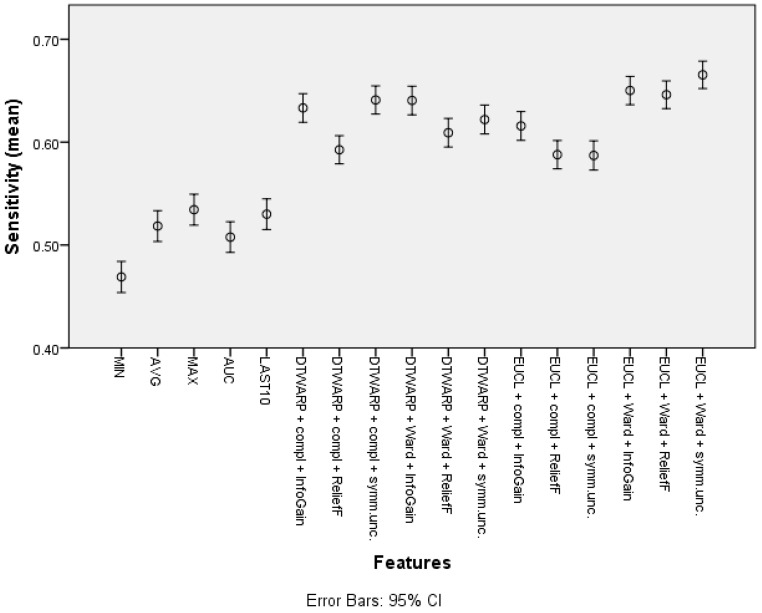
Sensitivity of Naïve Bayes classifiers: mean values and 95% confidence intervals.

The mean sensitivity (shown in Figure 6, with 95% confidence intervals as error bars) in the datasets with common features was between 46.9% (45.39–48.41%) and 53.44% (51.94–54.94%). The use of cluster taxonomies improved the mean sensitivity of Naïve Bayes models to 66.54% (65.21–67.87%). In this case, the best result was achieved in the dataset in which the clusters were obtained using Euclidean distance and Ward linkage, although there was no statistically significant difference from DTWARP combinations with complete linkage (InfoGain and symmetrical uncertainty feature selection) or with Ward linkage (InfoGain feature selection). The performance of the ReliefF feature selection was also worse when sensitivity was used as the metric for all of the combinations of distance measurements and linkage approaches.

**Figure 7:**
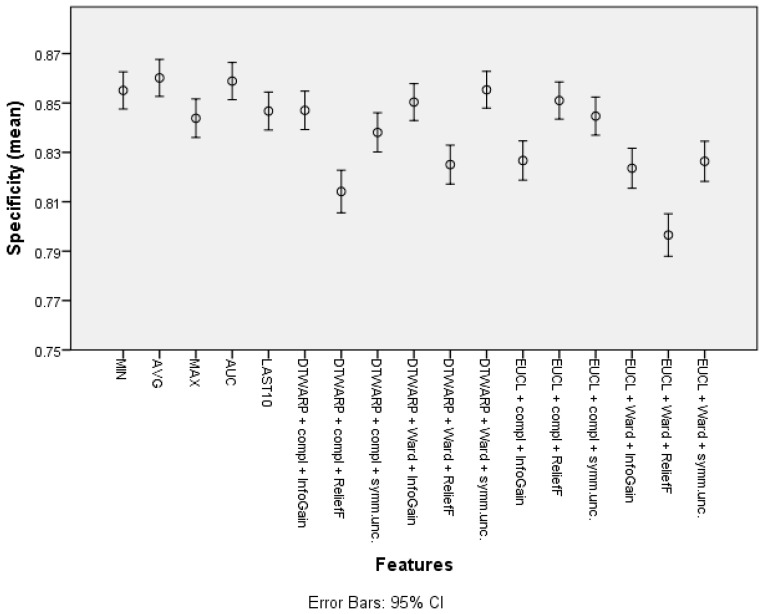
Specificity of Naïve Bayes classifiers: mean values and 95% confidence intervals.

The mean specificity (given in Figure 7, with 95% confidence intervals as error bars) in the datasets representing the common features of the sensor response curve was between 84.38% (83.6–85.16%) and 86.02% (85.27–86.76%). When cluster taxonomies were used instead of common features, the specificity decreased in most cases. The datasets in which the cluster cuts were obtained using ReliefF feature selection had the worst specificities, but InfoGain and symmetrical uncertainty provided good results, in which the difference from the specificities obtained using common features was 1–2 percentage points.

**Figure 8:**
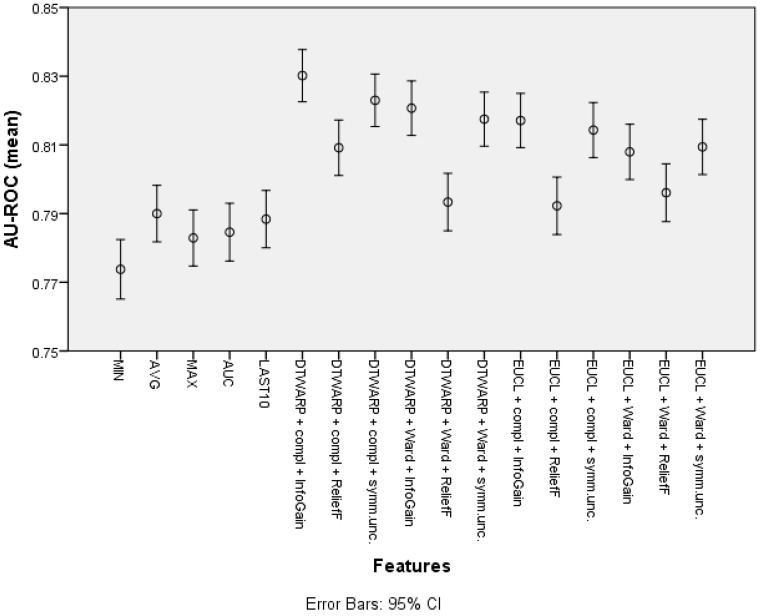
The area under the ROC curve of Naïve Bayes classifiers: mean values and 95% confidence intervals.

The mean area under the ROC curve (presented in Figure 8 with 95% confidence intervals as error bars) was lower in the datasets in which the common features were used; it ranged from 0.774 (0.765–0.782) to 0.790 (0.782–0.798). The AU-ROC was also lower in the datasets in which the cuts in taxonomies were chosen using ReliefF feature selection. The best result was obtained in the dataset in which the combination of DTWARP distance measurement and complete linkage was used together with InfoGain feature selection to select the best cuts in the cluster taxonomies; it was 0.830 (0.823–0.838). However, in most cases, the AU-ROC differences among the cluster taxonomy-based datasets were not statistically significant. Nevertheless, they differed significantly from the datasets in which the common features were used (except the cluster taxonomy-based datasets in which ReliefF was used to select the best cuts in the taxonomies).

**Figure 9:**
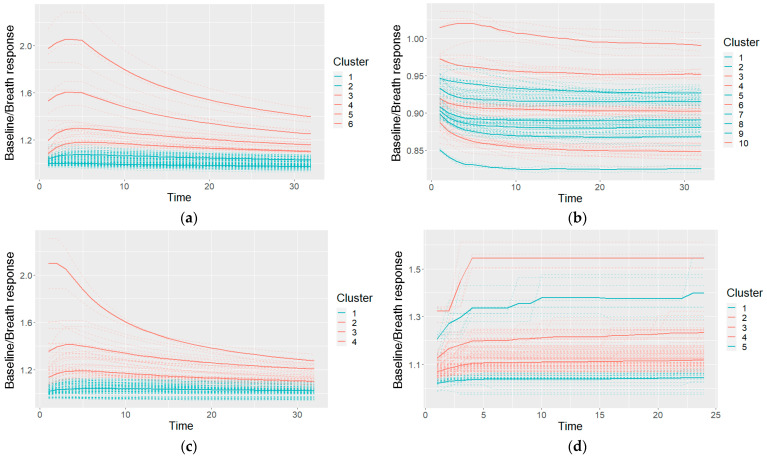
An example of the characteristic shapes used in a Naïve Bayes model: a taxonomy for GNP sensor responses cut at six clusters (**a**), taxonomies of two other GNP sensors cut at 10 and four clusters (**b**,**c**), and one MOXD sensor at five clusters (**d**); the dashed lines shows individual measurements, and the solid bold lines show the cluster-characteristic shapes.

An example of the clusters used in a Naïve Bayes classifier (after making cuts in the cluster taxonomies) and their characteristic shapes (the mean values of the clusters) is given in Figure 9. The shapes are colored on the basis of the probabilities of the cancer and control classes: if the model included a higher probability of cancer for the specific shape, it is colored red; otherwise (a higher probability of control class) it is colored blue.

**Figure 10:**
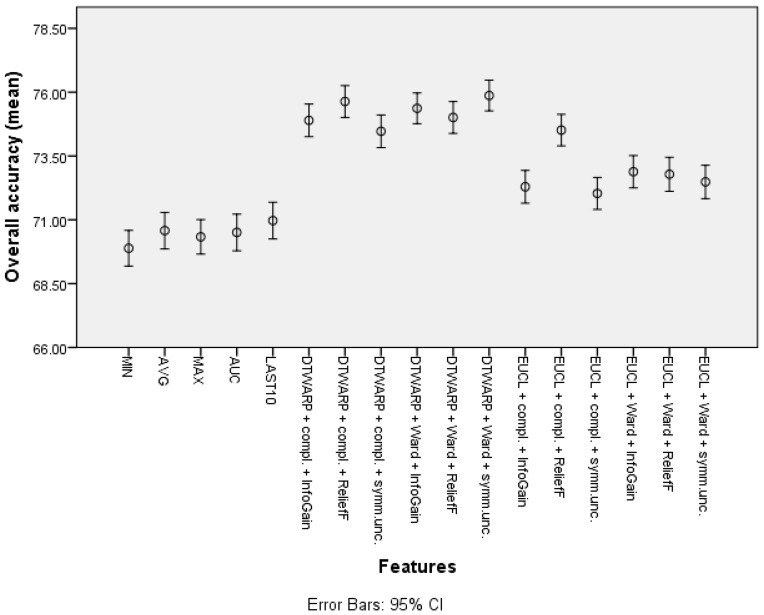
The overall accuracy of Random Forests: mean values and 95% confidence intervals.

The second-best results were acquired using Random Forests as classifiers (see the best results in Table 2; all of the accuracy metrics, including ROC, for all of the combinations are provided in Table S2 in the Supplementary Information). The overall accuracies (shown in Figure 10 with 95% CI as error bars) in the datasets with common features range from 69.89% (69.19–70.59%) to 70.97% (70.25–71.69%), and in the datasets in which the cluster taxonomies were used they range from 72.04% (71.41–72.66%) to 75.87% (75.27–76.47%). These results show great improvement, and the best result is not much worse than that of the Naïve Bayes classifier. However, the sensitivities acquired using RF are much inferior to those acquired using NB classifiers. The sensitivities in the datasets with common features range from 43.23% (41.82–44.64%) to 48.79% (47.32–50.25%), and in the cluster-based datasets they are between 39.59% (38.21–40.98%) and 46.52% (45.13–47.91%), much worse than the sensitivities obtained using NB classifiers. The relatively good overall accuracies are due to the higher specificities: from 82.78% (81.96–83.6%) to 84.72% (83.95–85.49%) in the datasets with common features, and from 86.93% (86.22–87.64%) to 92.39% (91.84–92.94%) in the cluster-based datasets. Like the overall accuracies, the AU-ROC results are also fairly good: from 0.763 (0.755–0.771) to 0.785 (0.777–0.793) in the datasets with common features, and from 0.745 (0.737–0.754) to 0.800 (0.792–0.808) in the cluster-based results.

If we look at the performances of different combinations of distance measures, linkage approaches and feature selection methods used to select the cuts in the cluster taxonomies, we can see that the results of the ReliefF feature selection approach are not worse, as in the case of NB. In some distance measurement and linkage approach combinations, they show the best results. Furthermore, when Random Forests are used as classifiers, there is no single best or worst feature selection method for all of the combinations. The overall accuracies obtained using DTWARP distance measurements and the choice of linkage approach do not seem to have a great influence. The situation is similar in the results obtained using other accuracy evaluation metrics.

The best results obtained using SVMs are presented in Table 3; see Table S3 in the Supplementary Information for the AU-ROC and results for other combinations of methods for clustering. The use of cluster taxonomies mainly affected the sensitivities of SVMs. The mean sensitivity for common features ranged from 40.86% (39.48–42.25%) to 48.33% (46.94–49.71%), and for clusters it was from 54.33% (52.9–55.76%) to 60.73% (59.31–62.15%). This means that SVMs suffered a loss in specificity when cluster taxonomies were used: the mean specificity for common features ranged from 88.2% (87.53–88.88%) to 91.14% (90.51–91.78%), while the use of cluster taxonomies resulted in specificities from 77.53% (76.63–78.43%) to 82.48% (81.66–83.30%).

**Figure 11:**
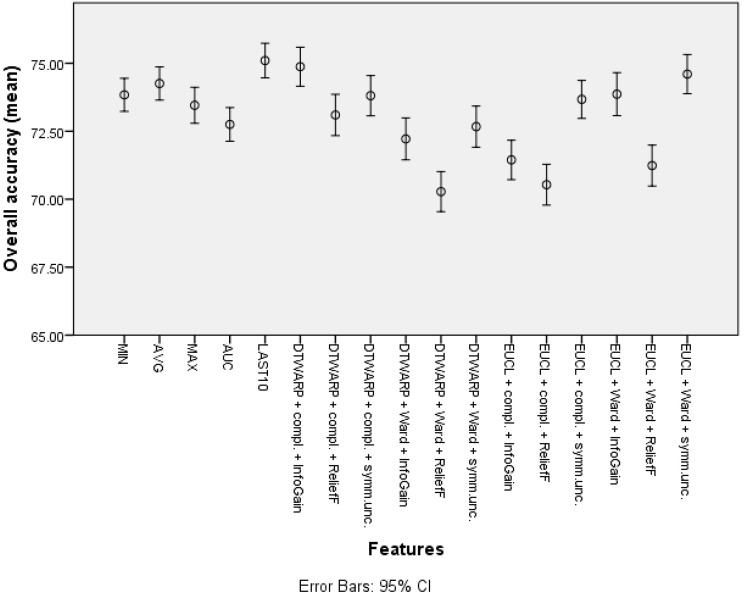
The overall accuracy of SVMs: mean values and 95% confidence intervals.

The overall accuracies (Figure 11) in this case were more similar among all of the features, ranging from 72.75% (72.13–73.37%) to 74.26% (73.64–74.87%) for common features, and from 70.28% (69.54–71.02%) to 74.87% (74.16–75.59%) for cluster-based datasets. Similar trends could be observed in AU-ROCs: from 0.653 (0.645–0.66) to 0.688 (0.68–0.696), and from 0.665 (0.657–0.674) to 0.716 (0.708–0.724), respectively.

## 4. Discussion

Breath analysis is a promising technique for the non-invasive early detection of cancer, and recent developments have shown that it can be applied to gastric cancer. During the past few decades, many types of sensors have been developed and implemented in laboratory-based settings, and there are reports of electronic nose systems being used in combination with data analysis methods for the rapid detection and clinical diagnosis of gastric cancer in an online mode (point-of-care devices). These would make breath analysis more accessible, and the detection process quicker [16,17,24,42,43,44,45,46].

Although many approaches and sensor materials have been designed and used to detect gastric cancer through exhaled breath, most of the previous solutions have been either technically complicated and relatively expensive, or insufficiently functional. Clinical diagnosis based on the detection of VOCs faces several challenges related to aspects of the sensor technology, mostly on the following major fronts: complexities of metabolism and VOC kinetics in a multianalyte system, the inter/intra-person variability of VOC profiles in such a complex environment, the standardization of sensor calibration owing to inherent sensor-to-sensor variability, and sensor drift and cross-sensitivities to environmental variables such as temperature. Such limitations of breath sensor technology are addressed in the current study: the proposed device includes not only GNP but also metal oxide sensors, which are selective for a number of VOCs and have been tested for their reproducibility and robustness.

In this article, we evaluated the diagnostic performance of a novel multi-sensor-based breath analyzer in gastric cancer patients. The device could differentiate between the breath prints of patients with gastric cancer and healthy controls with an overall accuracy of 77.8% (sensitivity: up to 66.54%; specificity: up to 92.39%). The specificities are higher so that these models can provide a better fit for screening applications, which should minimize the number of healthy patients having to undergo unnecessary invasive diagnostic procedures. Furthermore, the classification experiments with sensors of a single type showed worse results (especially sensitivity), indicating that additional modules with different sensors supplement the information and improve the differentiation.

The proposed novel machine learning approach, which applies taxonomies of sensor response curve shapes instead of single-point or estimate features, improves the accuracy of the classifiers obtained. However, it is important to choose the correct cuts in the cluster taxonomies (e.g., ReliefF in the case of NB). In some cases, the gain is lower than that in others; this is not a fit-for-all solution. Furthermore, the best methods for building taxonomies and classification can be different for different datasets and goals; therefore, it is necessary to apply several methods and compare their results in the actual dataset. The best results in this case were achieved using Naïve Bayes classifiers. Although RF provides good specificity, which is crucial for screening, its sensitivities are unacceptably low.

The overall results were better than those achieved by Schuermans et al. [47] using the Aeonose electronic nose to classify breath samples from a small group of gastric cancer patients and controls (81% sensitivity, 71% specificity, and 75% overall accuracy). Although some studies—e.g., [2,48]—report accuracies of over 90% (with sensitivity/specificity even as high as 100%), it is hard to compare them with the results of our study because they report only the results of one model/run of training, and some do not even use separate testing data.

## 5. Conclusions

The proposed device allows the breath to be analyzed in any location, thus providing more flexibility than laboratory-based approaches. It uses a combination of GNP and metal oxide sensors, and provides good and stable accuracy. Paired with the suggested data analysis methods, it can provide a quick and accurate technique for the detection of gastric cancer-specific breath, and potentially for other applications, e.g., the detection of other cancers, the monitoring of the course of diseases, and population-based screening.

## Figures and Tables

**Table 1:** Classification results (and 95%CI) using Naïve Bayes classifiers.

Feature	Overall Accuracy	Sensitivity	Specificity
Minimum	72.18% (71.49–72.87%)	46.9% (45.39–48.41%)	85.51% (84.76–86.26%)
Average	74.21% (73.5–74.91%)	51.85% (50.35–53.34%)	86.02% (85.27–86.76%)
Maximum	73.7% (72.96–74.44%)	53.44% (51.94–54.94%)	84.38% (83.6–85.16%)
Average of the last 10 time points	73.74% (73.02–74.45%)	53.00% (51.51–54.49%)	84.67% (83.9–85.44%)
Area under the curve	73.75% (73.04–74.47%)	50.77% (49.28–52.26%)	85.88% (85.13–86.64%)
Cluster (DTWARP distance, Ward linkage, InfoGain)	77.81% (77.15–78.48%)	64.05% (62.66–65.44%)	85.04% (84.29–85.78%)
Cluster (Euclidean distance, Ward linkage, Symm.Unc.)	77.1% (76.41–77.79%)	66.54% (65.21–67.87%)	82.64% (81.83–83.45%)

**Table 2:** Classification results using Random Forests.

Feature	Overall Accuracy	Sensitivity	Specificity
Minimum	69.89% (69.19–70.59%)	45.3% (43.87–46.73%)	82.93% (82.12–83.74%)
Average	70.58% (69.86–71.29%)	47% (45.56–48.45%)	83.13% (82.3–83.95%)
Maximum	70.33% (69.66–71.01%)	43.23% (41.82–44.64%)	84.72% (83.95–85.49%)
Average of the last 10 time points	70.97% (70.25–71.69%)	48.79% (47.32–50.25%)	82.78% (81.96–83.6%)
Area under the curve	70.51% (69.79–71.23%)	46.54% (45.1–47.99%)	83.27% (82.44–84.1%)
Cluster (DTWARP distance, Ward linkage, ReliefF)	75.01% (74.39–75.63%)	46.52% (45.13–47.91%)	90.12% (89.5–90.74%)
Cluster (Euclidean distance, complete linkage, ReliefF)	74.51% (73.90–75.13%)	46.04% (44.68–47.41%)	89.66% (89.01–90.30%)

**Table 3:** Classification results using SVMs.

Feature	Overall Accuracy	Sensitivity	Specificity
Minimum	73.84% (73.23–74.45%)	41.31% (39.99–42.64%)	91.14% (90.51–91.78%)
Maximum	73.45% (72.79–74.11%)	45.72% (44.31–47.14%)	88.2% (87.53–88.88%)
Average	74.26% (73.64–74.87%)	43.16% (41.77–44.55%)	90.74% (90.12–91.37%)
Average of the last 10 time points	75.1% (74.47–75.74%)	48.33% (46.94–49.71%)	89.27% (88.62–89.92%)
Area under the curve	72.75% (72.13–73.37%)	40.86% (39.48–42.25%)	89.68% (89.02–90.33%)
Cluster (DTWARP distance, complete linkage, InfoGain)	74.87% (74.16–75.59%)	60.73% (59.31–62.15%)	82.48% (81.66–83.30%)
Cluster (Euclidean distance, Ward linkage, InfoGain)	73.86% (73.07–74.65%)	61.05% (59.52–62.58%)	80.72% (79.84–81.6%)

## Data Availability

The data presented in this study are available on request from the corresponding author. They are not publicly available because of privacy and ethics considerations (regarding the study participants).

## Supplementary Materials

The following are available online at https://www.mdpi.com/article/10.3390/diagnostics12020491/s1, Table S1: Classification results (and 95% CI) using Naïve Bayes classifiers, Table S2: Classification results using Random Forests, Table S3: Classification results using SVMs.
