# Supplementary material for: Modular Point-of-Care Breath Analyzer and Shape Taxonomy-Based Machine Learning for Gastric Cancer Detection
Source: Diagnostics (Basel). 2022 Feb 14;12(2):491. doi: 10.3390/diagnostics12020491 (PMC8871298; doi:10.3390/diagnostics12020491)
Supplement: Supplementary file 1 [file diagnostics-12-00491-s001.zip › diagnostics-1572767-supplementary.pdf]

Supplementary information:

Classification results

The best classification results in this dataset were obtained using Naïve Bayes algorithm. The best overall accuracy was 77.81% and it was acquired in the data set, where cluster taxonomies were used; the taxonomies were built using DTWARP distance among curves, Ward linkage among clusters and Information Gain as the metric to select the best cut in the taxonomies. This combination also showed a good sensitivity (64.05%) and specificity (85.04%). The combination that identified cancer-specific breaths most accurately included Euclidean distance, Ward linkage and Symmetrical Uncertainty-based feature selection approach, identifying 66.54% of cancer-specific breaths correctly. The feature that allowed detecting the healthy participants' breaths most accurately was the average signal, however, several combinations of taxonomy-based classification approaches showed specificities above 85% and the differences in specificity here were not statistically significant. The best AU-ROC was also achieved using a taxonomy-based dataset using a combination of DTWARP distance, complete linkage and Information Gain metric. This means that with similar specificity the proposed approach improves overall accuracy, sensitivity and/or ROC.

Table S1. Classification results (and 95%CI) using Naïve Bayes classifiers.

Feature	Overall Accuracy	Sensitivity	Specificity	AU-ROC
Minimum	72.18% (71.49%…72.87%)	46.9% (45.39%…48.41%)	85.51% (84.76%…86.26%)	0.774 (0.765…0.782)
Average	74.21% (73.5%…74.91%)	51.85% (50.35%…53.34%)	86.02% (85.27%…86.76%)	0.790 (0.782…0.798)
Maximum	73.7% (72.96%…74.44%)	53.44% (51.94%…54.94%)	84.38% (83.6%…85.16%)	0.783 (0.775…0.791)
Average of the last 10 time points	73.74% (73.02%…74.45%)	53.00% (51.51%…54.49%)	84.67% (83.9%…85.44%)	0.788 (0.78…0.797)
Area under the curve	73.75% (73.04%…74.47%)	50.77% (49.28%…52.26%)	85.88% (85.13%…86.64%)	0.785 (0.776…0.793)
Cluster (DTWARP distance, complete linkage, InfoGain)	77.33% (76.62%...78.04%)	63.32% (61.93%...64.72%)	84.70% (83.93%...85.48%)	0.830 (0.823…0.838)
Cluster (DTWARP distance, complete linkage, ReliefF)	73.79% (73.06%...74.51%)	59.26% (57.88%...60.63%)	81.42% (80.55%...82.28%)	0.809 (0.801…0.817)
Cluster (DTWARP distance, complete linkage, Symm.Unc.)	77.01% (76.33%…77.70%)	64.10% (62.72%...65.47%)	83.81% (83.01%...84.60%)	0.823 (0.815…0.831)
Cluster (DTWARP distance, Ward linkage, InfoGain)	77.81% (77.15%…78.48%)	64.05% (62.66%…65.44%)	85.04% (84.29%…85.78%)	0.808 (0.8…0.816)
Cluster (DTWARP distance, Ward linkage, ReliefF)	75.07% (74.39%…75.75%)	60.92% (59.53%…62.3%)	82.5% (81.71%…83.29%)	0.796 (0.788…0.804)
Cluster (DTWARP distance, Ward linkage, Symm.Unc.)	77.5% (76.83%…78.18%)	62.2% (60.79%…63.6%)	85.53% (84.79%…86.28%)	0.809 (0.801…0.817)
Cluster (Euclidean distance, complete linkage, InfoGain)	75.40% (74.70%...76.09%)	61.58% (60.17%...62.98%)	82.67% (81.87%...83.46%)	0.817 (0.809…0.825)
Cluster (Euclidean distance, complete linkage, ReliefF)	76.03% (75.36%...76.70%)	58.79% (57.41%...60.16%)	85.10% (84.35%...85.85%)	0.792 (0.784…0.801)
Cluster (Euclidean distance, complete linkage, Symm.Unc.)	75.59% (74.89%…76.28%)	58.71% (57.29%...60.13%)	84.47% (83.69%...85.24%)	0.814 (0.806…0.822)
Cluster (Euclidean distance, Ward linkage, InfoGain)	76.39% (75.69%…77.1%)	65.02% (63.64%…66.4%)	82.36% (81.55%…83.17%)	0.821 (0.813…0.829)
Cluster (Euclidean distance, Ward linkage, ReliefF)	74.47% (73.76%…75.18%)	64.61% (63.26%…65.96%)	79.66% (78.8%…80.52%)	0.793 (0.785…0.802)
Cluster (Euclidean distance, Ward linkage, Symm.Unc.)	77.1% (76.41%…77.79%)	66.54% (65.21%…67.87%)	82.64% (81.83%…83.45%)	0.817 (0.81…0.825)

The second-best classification results in this dataset were obtained using Random Forest algorithm. The best overall accuracy was 75.87%, obtained using the data set, where cluster taxonomies were used; the taxonomies were built using DTWARP distance among curves, Ward linkage among clusters and Symmetrical uncertainty as the metric to select the best cut in the taxonomies. All of the feautes and approaches showed poor sensitivity (less than 50%). However, specificity was significantly improved using taxonomies, reaching up to 92.39% specificity for the same combination that showed the best overall accuracy. The two best AU-ROCs (0.800 and 0.799) were acquired using taxonomies: Euclidean distance, complete linkage, ReliefF-based feature selection, and DTWARP distance, complete linkage, ReliefF-based feature selection respectively.

Table S2. Classification results using Random Forests.

Feature	Overall Accuracy	Sensitivity	Specificity	AU-ROC
Minimum	69.89% (69.19%…70.59%)	45.3% (43.87%…46.73%)	82.93% (82.12%…83.74%)	0.763 (0.755…0.771)
Average	70.58% (69.86%…71.29%)	47% (45.56%…48.45%)	83.13% (82.3%…83.95%)	0.781 (0.773…0.789)
Maximum	70.33% (69.66%…71.01%)	43.23% (41.82%…44.64%)	84.72% (83.95%…85.49%)	0.771 (0.762…0.779)
Average of the last 10 time points	70.97% (70.25%…71.69%)	48.79% (47.32%…50.25%)	82.78% (81.96%…83.6%)	0.785 (0.777…0.793)
Area under the curve	70.51% (69.79%…71.23%)	46.54% (45.1%…47.99%)	83.27% (82.44%…84.1%)	0.781 (0.773…0.789)
Cluster (DTWARP distance, complete linkage, InfoGain)	74.90% (74.26%...7.54%)	45.77% (44.38%...47.17%)	90.37% (89.72%...91.02%)	0.783 (0.775…0.791)
Cluster (DTWARP distance, complete linkage, ReliefF)	75.64% (75.01%...76.26%)	45.95% (44.56%...47.34%)	91.39% (90.80%...91.99%)	0.799 (0.792…0.807)
Cluster (DTWARP distance, complete linkage, Symm.Unc.)	74.47% (73.83%...75.11%)	45.98% (44.60%...47.36%)	89.58% (88.91%...90.24%)	0.786 (0.778…0.794)
Cluster (DTWARP distance, Ward linkage, InfoGain)	75.37% (74.76%…75.97%)	44.38% (42.99%…45.77%)	91.79% (91.21%…92.38%)	0.787 (0.778…0.795)
Cluster (DTWARP distance, Ward linkage, ReliefF)	75.01% (74.39%…75.63%)	46.52% (45.13%…47.91%)	90.12% (89.5%…90.74%)	0.761 (0.752…0.769)
Cluster (DTWARP distance, Ward linkage, Symm.Unc.)	75.87% (75.27%…76.47%)	44.69% (43.31%…46.07%)	92.39% (91.84%…92.94%)	0.777 (0.769…0.786)
Cluster (Euclidean distance, complete linkage, InfoGain)	72.29% (71.65%...72.94%)	40.57% (39.20%...41.95%)	89.18% (88.52%...89.84%)	0.792 (0.784…0.800)
Cluster (Euclidean distance, complete linkage, ReliefF)	74.51% (73.90%...75.13%)	46.04% (44.68%...47.41%)	89.66% (89.01%...90.30%)	0.800 (0.792…0.808)
Cluster (Euclidean distance, complete linkage, Symm.Unc.)	72.04% (71.41%...72.66%)	39.59% (38.21%...40.98%)	89.27% (88.61%...89.93%)	0.791 (0.783…0.799)
Cluster (Euclidean distance, Ward linkage, InfoGain)	72.88% (72.25%…73.52%)	42.57% (41.18%…43.96%)	89.02% (88.36%…89.69%)	0.75 (0.741…0.759)
Cluster (Euclidean distance, Ward linkage, ReliefF)	72.78% (72.12%…73.45%)	46.13% (44.75%…47.52%)	86.93% (86.22%…87.64%)	0.747 (0.738…0.756)
Cluster (Euclidean distance, Ward linkage, Symm.Unc.)	72.49% (71.83%…73.14%)	44.57% (43.17%…45.98%)	87.33% (86.63%…88.03%)	0.745 (0.737…0.754)

The third-best classification results in this dataset were obtained using SVM algorithm. The best overall accuracy was 75.10%, obtained using the data set, where cluster taxonomies were not applied. The data set used the average signal value of the last 10 time-points of the curve. The second-best result (74.87%) was acquired in the data set that used DTWARP distance, complete linkage and Information Gain metric for feature selection. The best sensitivity (61.05%) was acquired in the data set, where the combination of Euclidean distance, Ward linkage and Information Gain metric for feature selection. Specificity was lower in the data sets where taxonomies were used, and the best specificity (91.14%) was obtained using the minimum value of the sensor response curve, however sensitivity in this case was unacceptably low (41.31%). The best AU-ROC (0.716) was acquired using taxonomies: DTWARP distance, complete linkage and Information Gain for feature selection.

Table S3. Classification results using SVMs.

Feature	Overall Accuracy	Sensitivity	Specificity	AU-ROC
Minimum	73.84% (73.23%…74.45%)	41.31% (39.99%…42.64%)	91.14% (90.51%…91.78%)	0.662 (0.655…0.669)
Maximum	73.45% (72.79%…74.11%)	45.72% (44.31%…47.14%)	88.2% (87.53%…88.88%)	0.67 (0.662…0.677)
Average	74.26% (73.64%…74.87%)	43.16% (41.77%…44.55%)	90.74% (90.12%…91.37%)	0.653 (0.645…0.66)
Average of the last 10 time points	75.1% (74.47%…75.74%)	48.33% (46.94%…49.71%)	89.27% (88.62%…89.92%)	0.688 (0.68…0.696)
Area under the curve	72.75% (72.13%…73.37%)	40.86% (39.48%…42.25%)	89.68% (89.02%…90.33%)	0.67 (0.662…0.677)
Cluster (DTWARP distance, complete linkage, InfoGain)	74.87% (74.16%...75.59%)	60.73% (59.31%...62.15%)	82.48% (81.66%...83.30%)	0.716 (0.708…0.724)
Cluster (DTWARP distance, complete linkage, ReliefF)	73.10% (72.34%...73.86%)	59.66% (58.15%...61.16%)	80.34% (79.45%...81.22%)	0.700 (0.691…0.709)
Cluster (DTWARP distance, complete linkage, Symm.Unc.)	73.81% (73.07%...74.55%)	58.13% (56.69%...59.56%)	82.18% (81.32%...83.05%)	0.702 (0.693…0.710)
Cluster (DTWARP distance, Ward linkage, InfoGain)	72.22% (71.45%…72.99%)	58.25% (56.76%…59.74%)	79.71% (78.83%…80.59%)	0.69 (0.681…0.698)
Cluster (DTWARP distance, Ward linkage, ReliefF)	70.28% (69.54%…71.02%)	54.33% (52.9%…55.76%)	78.76% (77.85%…79.67%)	0.665 (0.657…0.674)
Cluster (DTWARP distance, Ward linkage, Symm.Unc.)	72.67% (71.91%…73.42%)	58.46% (56.99%…59.92%)	80.28% (79.43%…81.13%)	0.694 (0.685…0.702)
Cluster (Euclidean distance, complete linkage, InfoGain)	71.45% (70.72%...72.17%)	55.94% (54.48%...57.39%)	79.76% (78.89%...80.62%)	0.679 (0.670…0.687)
Cluster (Euclidean distance, complete linkage, ReliefF)	70.53% (69.78%...71.28%)	57.50% (56.00%...58.99%)	77.53% (76.63%...78.43%)	0.675 (0.667…0.684)
Cluster (Euclidean distance, complete linkage, Symm.Unc.)	73.67% (72.97%...74.37%)	58.21% (56.80%...59.62%)	81.92% (81.08%...82.77%)	0.701 (0.693…0.709)
Cluster (Euclidean distance, Ward linkage, InfoGain)	73.86% (73.07%…74.65%)	61.05% (59.52%…62.58%)	80.72% (79.84%…81.6%)	0.709 (0.7…0.718)
Cluster (Euclidean distance, Ward linkage, ReliefF)	71.24% (70.49%…71.99%)	55.58% (54.12%…57.03%)	79.58% (78.69%…80.46%)	0.676 (0.667…0.684)
Cluster (Euclidean distance, Ward linkage, Symm.Unc.)	74.6% (73.88%…75.32%)	59.97% (58.52%…61.41%)	82.41% (81.59%…83.23%)	0.712 (0.704…0.72)
